# Comparative analysis of butternut (*Juglans cinerea*) and Japanese walnut (*Juglans ailantifolia*) chloroplast genomes

**DOI:** 10.1186/s12870-025-07678-1

**Published:** 2025-12-08

**Authors:** Aziz Ebrahimi, Mojtaba Zamani Faradonbeh, Abdullah, Samarth Mathur, Martin Williams, Anna O. Conrad, Douglass F. Jacobs

**Affiliations:** 1 Department of Forestry and Natural Resources, Purdue University, West Lafayette, IN USA; 2 State Key Laboratory of Chinese Medicine Modernization, Tianjin University of Traditional Chinese Medicine, Tianjin, 301617 China; 3 Haihe Laboratory of Modern Chinese Medicine, Tianjin, 301617 China; 4 Department of Evolution, Ecology, and Organismal Biology, The Ohio State University, Columbus, OH USA; 5 Natural Resources Canada, Canadian Forest Center, Atlantic Forestry Center, 1350 Regent Street, Fredericton, NB E3C 2G6 Canada; 6 USDA Forest Service, Northern Research Station, Delaware, OH 43015 USA

**Keywords:** Butternut, Conservation genetics, Species-specific CAPS markers, Genetic diversity, Comparative genome, Species identification

## Abstract

*Juglans cinerea* L. (2n = 16), commonly known as butternut, is a diploid species native to the eastern forests of the United States and Canada. It plays a crucial ecological role as a food source for wildlife and is traditionally used as a medicine by tribal communities. Butternut populations have severely declined over 50 years due to butternut canker disease (BCD). The ability of butternut to hybridize with Japanese walnut, which exhibits resistance to BCD, complicates hybrid identification based on morphological traits alone. In this study, we assembled the chloroplast genome of butternut using next-generation sequencing, resulting in a genome size of 160,289 bp. The genome comprises 112 genes, including 78 protein-coding genes, 30 tRNA genes, and four rRNA genes, with a GC content of 36%. Comparative analysis revealed 1,156 SNPs, including 500 species-specific SNPs between 13 butternut and 6 Japanese walnut accessions. Of these, 367 SNPs were in coding regions, 32 of which exhibited unique variations causing codon and amino acid changes between the two species. We developed and validated 12 species-specific CAPS markers in *matK* and *ycf1* genes for assessing the genetic background of *Juglans* species. The *ycf1* gene displayed the highest variability, harboring 37 unique SNPs, many resulting in non-synonymous mutations. The newly sequenced chloroplast genome of *J. cinerea* and the identified SNP resources provide valuable genetic tools for characterizing butternut’s genetic diversity and distinguishing it from Japanese walnut. These findings enhance our understanding of *Juglans* species and contribute significantly to plant genetics and genomics by comprehensively analyzing chloroplast genomes and SNP variation, addressing a critical gap in previous studies.

**Supplementary Information:**

The online version contains supplementary material available at 10.1186/s12870-025-07678-1.

## Introduction

*Juglans cinerea* L. (2n = 16), commonly known as butternut, is a diploid species native to the eastern United States and Canada. This species produces valuable timber for industry and plays an important ecological role by providing a rich food source for wildlife. Additionally, butternuts hold significant cultural importance for First Nations and tribal communities, who have used them as a food source, in medicine, and for artwork [1]. Unfortunately, like many North American forest trees (such as elm, ash, and chestnut), butternut populations have declined due to an invasive pathogen. Butternut canker disease (BCD), caused by the fungus *Ophiognomonia clavigignenti-juglandacearum*, was first identified in the late 1960s [2] and has since been detected across the entire range of the species. BCD infections have led to *J. cinerea* being listed as globally endangered, including in Canada [3, 4], and at risk in many U.S. states. With no cure available and minimal evidence of resistance within the species, efforts are ongoing in Canada and the U.S. to develop strategies for protecting North American butternut from extinction.

One promising approach involves taking advantage of naturally occurring butternut hybrids with Japanese walnut, which was introduced as an ornamental plant in the late 1800s. Butternut and Japanese walnut hybridize naturally in contact zones, producing offspring with varying levels of resistance to BCD. However, because butternut and Japanese walnut hybridize so readily, distinguishing between pure species and hybrids based on morphology alone can be challenging. Successful conservation and restoration of butternut will require precise genetic characterization of individual trees for future breeding efforts. Understanding the genetic background of these trees at both the nuclear and chloroplast levels is essential for identifying pure butternut from hybrids, particularly for advanced backcrosses (those with more than 90% butternut genome). Due to their maternal inheritance, the chloroplast genome can screen the maternal background of butternuts and their hybrids, helping to characterize the ancestral hybridization between butternuts and other *Juglans* species.

Presently, efforts are ongoing to conserve and restore this species, which requires a greater understanding of butternut and hybrid trees’ genetic background (i.e., population structure, gene flow, and marker-assisted breeding) to characterize their hybrid status and screen for resistant traits. Numerous studies have used nuclear-genome to evaluate the genetic diversity of *Juglans* species [5, 6]. Sequence data from chloroplast genomes have been used extensively in plant biology, including phylogenetic analysis [7, 8], biotechnological applications [9], and species identification [5, 10]. While structurally conserved, chloroplast genomes contain highly polymorphic regions facilitating accurate species identification [11, 12]. Additionally, their high copy number enhances the robustness of barcode region amplification. Chloroplast genome divergence is not high within species but higher at an interspecies-specific level [13]. Although useful for species identification, the genome’s maternal inheritance needs to be considered since chloroplast capture events can happen during hybridization between closely related congeners, which could lead to misidentification.

Several studies have shown that many chloroplast genes have been lost throughout an evolutionary event in the history of plants or have been functionally transferred to the nuclear genome [13]. For example, genes such as *tuf*A, *fts*H, and *odp*B have moved from the chloroplast to the nuclear genome in certain plant species [13]. Conversely, genes like *ycf1* have been reportedly lost in grasses and cranberries (Ericaceae) despite their essential roles in the import of TOC (translocon on the outer envelope of chloroplasts) and TIC (translocon on the inner envelope of chloroplasts) [14]. These gene transfers have resulted in the expansion, contraction, or even loss of genetic content and a decrease in the size of the chloroplast genome, with these evolutionary events being either species-specific or, in some cases, affecting entire plant orders [15–17]. Additionally, *ycf1* was the first plastid-encoded protein identified as essential for the survival of *Chlamydomonas reinhardtii* and tobacco (*Nicotiana tabacum*), although its specific function remained unclear at the time [14]. Due to its interspecific variation, *ycf1* is currently listed as one of land plants’ most promising plastid DNA barcodes compared to *matK* and *rbcL* [18], which are used for species identification. Using species-specific markers is a practical approach for accurate species identification and population genetic studies. This strategy enhances resolution in distinguishing closely related taxa and assessing genetic diversity within populations [19]. However, a thorough comparative analysis of the chloroplasts between the North American butternut species and its more disease-resistant Asian hybrid counterpart, the Japanese walnut, has yet to be performed and may provide a useful tool for species identification.

To better understand the maternal lineage and distinguish North American butternut from its Asian hybrid congener, we employed chloroplast genome analysis, which offers a high-resolution tool for species identification. Among molecular techniques, Cleaved Amplified Polymorphic Sequence (CAPS) markers, derived from fixed nucleotide differences that create or abolish restriction enzyme sites, are particularly useful for developing reliable, low-cost diagnostic tools. Building on this context, we structured our study around four main objectives: (1) assemble and characterize the complete chloroplast genome of butternut, (2) evaluate intraspecific SNP variation within each species and interspecific SNP variation between species, (3) investigate whether coding region variation may lead to changes at the protein level, and (4) design CAPS markers from fixed interspecific variation for use as a species identification and taxonomic barcoding tool. This study provides a reference chloroplast genome for butternut and identifies Coding DNA Sequence (CDS) derived SNP markers that differentiate the two species based on codon and amino acid variations. These CAPS markers, which have been developed, can be used as barcoding markers to determine the maternal lineage of hybrids, study population genetics, and conduct phylogenetic analyses.

## Materials and methods

### Chloroplast assembly of butternut

**Fig. 1:**
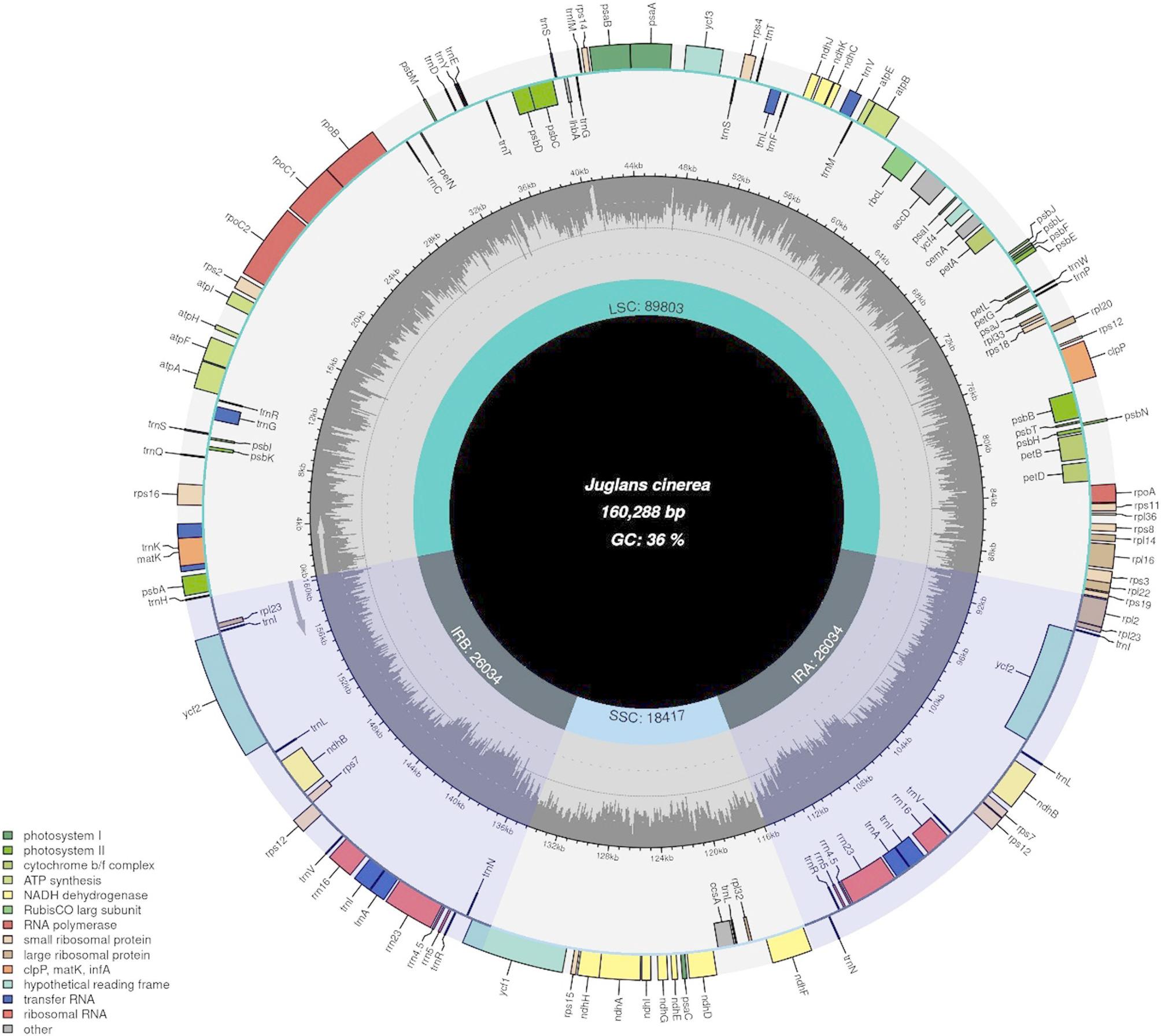
A circular map displaying the *Juglans cinerea* chloroplast genome with all the genes annotated. The genes transcribed clockwise are shown within the circle, while those transcribed anticlockwise are shown outside. LSC, SSR, IRA, and IRB define the borders of the chloroplast genome. Each gene is coded according to its specific function. A dashed grey color represents the inner circle showing GC content, while the lighter grey represents the AT range

Fresh butternut leaves (*accession #1351*) were collected from the butternut orchard at Purdue University, West Lafayette, Indiana, USA. DNA was extracted using the Doyle and Doyle method [20] with minor modifications. Whole-genome sequencing was performed using the Illumina short-read technology with paired-end libraries on the HiSeq 2500 sequencing platform in a single lane as described in Ebrahimi et al. [21]. The chloroplast *de-novo* genome assembly was completed using the GetOrganelle version (1.7.7.0) [22], and the assembled chloroplast was deposited in NCBI (*accession* # OR134831) (Fig. 1) by generating a feature table and related files for submission through GB2Sequin [23]. The genes in the chloroplast genome were predicted using GeSeq Annotation of Organellar Genomes [24], and the chloroplast map was drawn using Chloroplot [25]. To analyze the loss or presence/absence of genes in butternut (*J. cinerea*, Accession #OR134831) compared with Japanese walnut (*J. ailantifolia*, accession #NC_046433.1) and black walnut (*Juglans nigra* L., accession #NC_035967), we use the Feature Tables downloaded from NCBI and the presence/absence genes listed in Supplementary Tables 1 and MAFFT alignment of the mentioned species [26].

### Phylogeny analysis

**Fig. 2:**
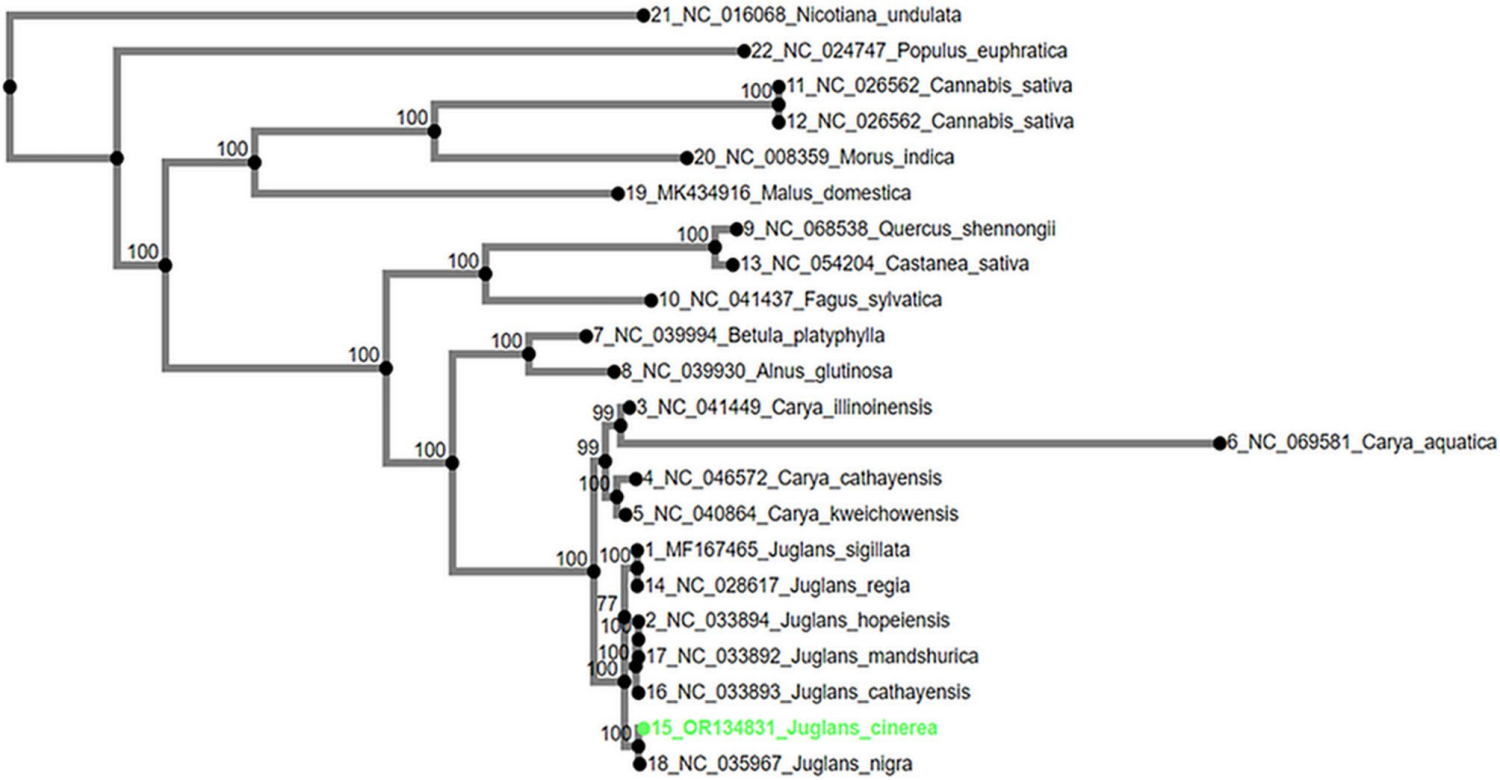
Maximum likelihood phylogenetic tree based on complete chloroplast genomes of *Juglans cinerea* and related species, aligned using MAFFT. Bootstrap values from 1,000 replicates are shown at the nodes. The tree includes *J. cinerea* (OR134831), four other *Juglans* species, and chloroplast genomes from related species in the Fagales and Rosales orders for comparative analysis

The chloroplast genomes of various *Juglans* (walnuts) and *Carya* (hickories) species were downloaded from NCBI. This includes the assembled chloroplast genome of butternut (*J. cinerea*, #OR134831). Phylogenetic analysis was conducted using the Maximum Likelihood analysis in RAxML, implemented in the MAFFT [16]. Bootstrap resampling was set to 1000 to ensure robust results. The phylogenetic trees and data were visualized using the tools available at ngphylogeny.fr and iTOL (Interactive Tree of Life). To conduct this analysis, we included the chloroplast genome of *J. cinerea* (accession number: OR134831) alongside four complete chloroplast genomes of other *Juglans* species. Additionally, chloroplast genome sequences from various other plant species were included for comparative purposes, such as *Nicotiana undulata* (tobacco, NC016068), *Cannabis sativa* (hemp, NC026562), *Morus indica* (mulberry, NC008359), *Populus euphratica* (Euphrates poplar, NC024747), *Castanea sativa* (sweet chestnut, NC054204), *Malus domestica* (apple, MK_434916), *Alnus glutinosa* (black alder, NC_039930.1), *Betula platyphylla* (white birch, NC_039994.1), *Carya aquatica* (water hickory, NC_069581.1), *Carya cathayensis* (Chinese hickory, NC_046572.1), *Carya illinoinensis* (pecan, NC_041449.1), *Carya kweichowensis* (Guizhou hickory, NC_040864.1), *Fagus sylvatica* (European beech, NC_041437.1), and *Quercus shennongii* (Shennong oak, NC_068538.1). This broad selection of species allowed for a thorough phylogenetic analysis (Fig. 2), helping to clarify the evolutionary relationships within and between the *Juglans* and *Carya* genera and other related species.

### Variant calling

**Fig. 3:**
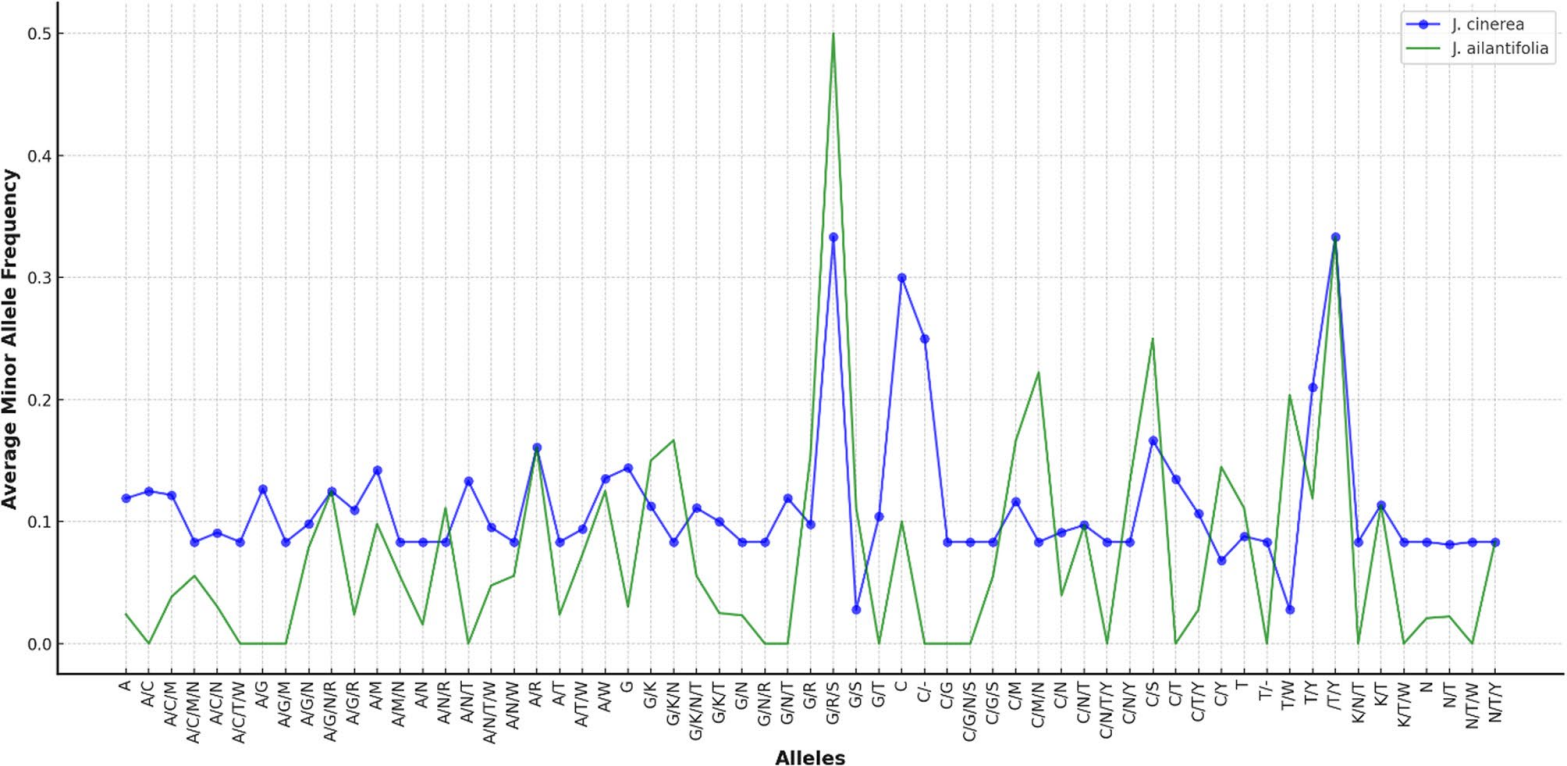
Comparison of minor Allele frequencies for *J. cinerea* and *J. ailantifolia*. The blue circles represent data for *J. cinerea*, while the green crosses correspond to *J. ailantifolia*. Alleles are arranged starting with A, G, C, and T, followed by alphabetical order for the remaining alleles

Paired-end sequences of 13 *J. cinerea* and 6 *J. ailantifolia* accessions were downloaded from NCBI (deposited by references 8, 25). Then, reads were aligned with the *J. cinerea* reference chloroplast genome. We processed the reads using BWA-MEM [27] for alignment and Picard tools (https://github.com/broadinstitute/picard) to remove duplicates [28]. We used GATK [29] for genotyping, applying quality filters to minimize errors and converting the *.hmp format (Supplementary Table 2). The genotype frequencies of A, T, G, C, and InDel (Insertion–Deletion) for each variants, as extracted using vcftools, are listed in Fig. 3 and Supplementary Table 5. Based on gene annotation results, the CDS regions start and stop positions of each gene was extracted, and each CDS SNP variant was identified in the *.vcf file using vcftools (Supplementary Table 3). Coding region SNPs were screened to identify unique variants specific to each species and subsequently analyzed to determine if these variants impacted codons and subsequent amino acid substitutions (Table 1, Supplementary Table 4).

**Table 1:** Summary of SNPs and unique variations across genome and coding region sequences of *MatK* and *ycf1* for butternut (JC) and Japanese walnut (JA) used for evaluating codon and amino acid variations (The information related to other genes listed in supplementary table 3). In the “amino acid type based on R group: JA-JC” column, N, P, A, B, and S are abbreviations for Nonpolar, Polar, Acidic, Basic, and Synonymous, respectively

ID	Total SNPs	SNPs unique	Unique SNP positions: genome	Unique SNP positions: CDS	JA/JC	Codon in JA	Codon in JC	Amino acid: JA/JC	Amino acid type based on *R* group: JA-JC*
*matK*	40	5	2069	1398	C/T	GAG	GAA	Glu/Glu	S
			2206	1261	G/T	CAA	AAA	Gln/Lys	P-B
			2801	666	G/T	TTC	TTA	Phe/Leu	N-N
			3177	290	C/A	GGA	GTA	Gly/Val	N-N
			3350	117	C/T	AGG	AGA	Arg/Arg	S
*ycf1*	84	37	129,813	5671	C/A	GAT	TAT	Asp/Tyr	A-P
			130,038	5446	G/A	CAT	TAT	His/Tyr	B-P
			130,126	5358	A/C	TTT	TTG	Phe/Leu	N-N
			130,600	4884	T/G	AAA	AAC	Lys/Asn	B-P
			130,656	4828	T/G	AAA	CAA	Lys/Gln	B-P
			130,733	4751	G/T	ACT	AAT	Thr/Asn	P-P
			130,744	4740	G/C	TCC	TCG	Ser/Ser	S
			130,783	4701	G/T	AGC	AGA	Ser/Arg	P-B
			130,847	4637	A/G	GTC	GCC	Val/Ala	N-N
			131,446	4038	C/T	ATG	ATA	Met/Ile	N-N
			131,584	3900	A/G	TCT	TCC	Ser/Ser	S
			131,825	3659	C/T	TGT	TAT	Cys/Tyr	P-P
			131,995	3489	A/T	TTT	TTA	Phe/Leu	N-N
			132,095	3389	G/A	TCA	TTA	Ser/Leu	P-N
			132,259	3225	A/G	ATT	ATC	Ile/Ile	S
			132,282	3202	T/G	ATA	CTA	Ile/Leu	N-N
			132,387	3097	T/C	AAA	GAA	Lys/Glu	B-A
			132,833	2651	G/C	ACA	AGA	Thr/Arg	P-B
			132,976	2508	T/A	ATA	ATT	Ile/Ile	S
			133,011	2473	G/T	CTT	ATT	Leu/Ile	N-N
			133,120	2364	T/G	AGA	AGC	Arg/Ser	B-P
			133,170	2314	G/A	CAT	TAT	His/Tyr	B-P
			133,192	2292	C/A	AAG	AAT	Lys/Asn	B-B
			133,287	2197	G/C	CCC	GCC	Pro/Ala	N-N
			133,616	1868	C/G	GGA	GCA	Gly/Ala	N-N
			133,681	1803	A/C	TGT	TGG	Cys/Trp	P-N
			133,710	1774	G/T	CTT	ATT	Leu/Ile	N-N
			133,756	1728	T/G	TTA	TTC	Leu/Phe	N-N
			133,770	1714	A/G	TCA	CCA	Ser/Pro	P-N
			133,787	1697	A/C	ATC	AGC	Ile/Ser	N-P
			133,906	1578	A/C	TTT	TTG	Phe/Leu	N-N
			133,942	1542	A/C	ACT	ACG	Thr/Thr	S
			134,109	1375	G/A	CTA	TTA	Leu/Leu	S
			134,137	1347	A/G	AAT	AAC	Asn/Asn	S
			134,304	1180	T/G	AAA	CAA	Lys/Gln	B-P
			134,334	1150	T/C	AAA	GAA	Lys/Glu	B-A
			134,374	1110	T/G	CGA	CGC	Arg/Arg	S

### Identification of species-specific SNPs and prediction of CAPS marker candidates

Species-specific SNPs within the *matK* and *ycf1* genes were identified from the chloroplast genomes of *Juglans* species. To assess whether these SNPs could be targeted with CAPS markers, we analyzed the surrounding sequences using the web-based CAPS Finder 2.0 (http://helix.wustl.edu/CAPS/). NEBcutter 2.0 (http://tools.neb.com/NEBcutter) was subsequently employed to examine restriction enzyme recognition sites within the amplified regions. Primers were then designed to amplify SNP-containing segments using the PrimerQuest™ Tool (https://www.idtdna.com/pages/tools/primerquest), ensuring that the predicted restriction fragments would be detectable by standard agarose gel electrophoresis.

### Validation of CAPS and dCAPS markers by PCR and restriction enzyme

The primers (Supplementary Table 6) were synthesized by Integrated DNA Technologies (IDT). PCR amplifications used GoTaq^®^ Green Master Mix (cat. no. M7123; Promega). The annealing temperature (Tm) was 57 °C for all primer pairs, except for marker CPS03-*ycf1*, which was amplified at 54 °C. Reactions were conducted in 25 µL volumes containing 20 ng of template DNA, with this program: initial denaturation at 94 °C for 3 min; 33 cycles of 94 °C for 45 s, annealing at the specified Tm for 45 s, and extension at 72 °C for 60 s; then a final extension at 72 °C for 5 min. Restriction enzymes (Supplementary Table 6) were purchased from New England Biolabs (NEB). For digestion, 10 µL of PCR product was used in a 30-µL reaction volume, following the manufacturer’s instructions for each enzyme with minor modifications, and the digestion time was increased to 60 min. Digested products were analyzed on 3% agarose gels to examine the fragmentation patterns.

## Results

### Chloroplast assembly of butternut

Paired-end genome data were used for the de novo assembly of the butternut chloroplast genome. The chloroplast genome comprises 112 genes, including 78 protein-coding genes, 30 tRNA genes, and four rRNA genes. The GC content is 36%, while the LSC length is 89,805 bp, the IR length is 26,035 bp, and the SSC length is 18,415 bp. The complete chloroplast genome length was 160,288 bp (Fig. 1). Comparative analysis revealed similar gene content in both butternut and black walnut after careful inspection of all the annotations and conducting a comparative study based on their alignment. The *infA* and *ycf15* were found as pseudogenes in all three species.

### Phylogenomic analysis of *Juglans* species

Phylogenomic analysis revealed that *J. cinerea* is closely related to *J. nigra*, while other *Juglans* species from Asia clustered together (Fig. 2). All *Juglans* and *Carya* species formed a monophyletic group consistent with their classification in the Juglandaceae family. The chloroplast genome of the Juglandaceae family was found to be more closely related to those of the *Alnus* and *Betula* genera than to those of the more distantly related *Fagus*, *Castanea*, and *Quercus* genera.

### SNPs in the chloroplast genome of butternut and Japanese walnut

We identified 1,156 SNPs in the chloroplast genomes of 13 butternut and 6 Japanese walnut samples (Supplementary Table 2). Among these, 366 SNPs were located in coding regions (Supplementary Table 3). Most SNPs in these coding regions belong to *ycf1* and *matK* genes, with 84 and 40 SNPs, respectively. Other notable SNP counts include 27 in *psa*A, 19 in *rpo*C2, and 17 in *rbc*L. Among the 47 CDS genes identified, *ycf1* and *matK* were the largest. Of these 47 genes, 32 exhibited both synonymous and non-synonymous SNP variations, while the remaining 15 CDS were not species-specific (Table 1; Supplementary Table 4). When comparing allele frequencies across all SNPs, we observed that the A, G, and C allele frequencies were higher in butternut than in Japanese walnut (Fig. 3).

### Codon and amino acid variations in chloroplast genomes of butternut and Japanese walnut

**Fig. 4:**
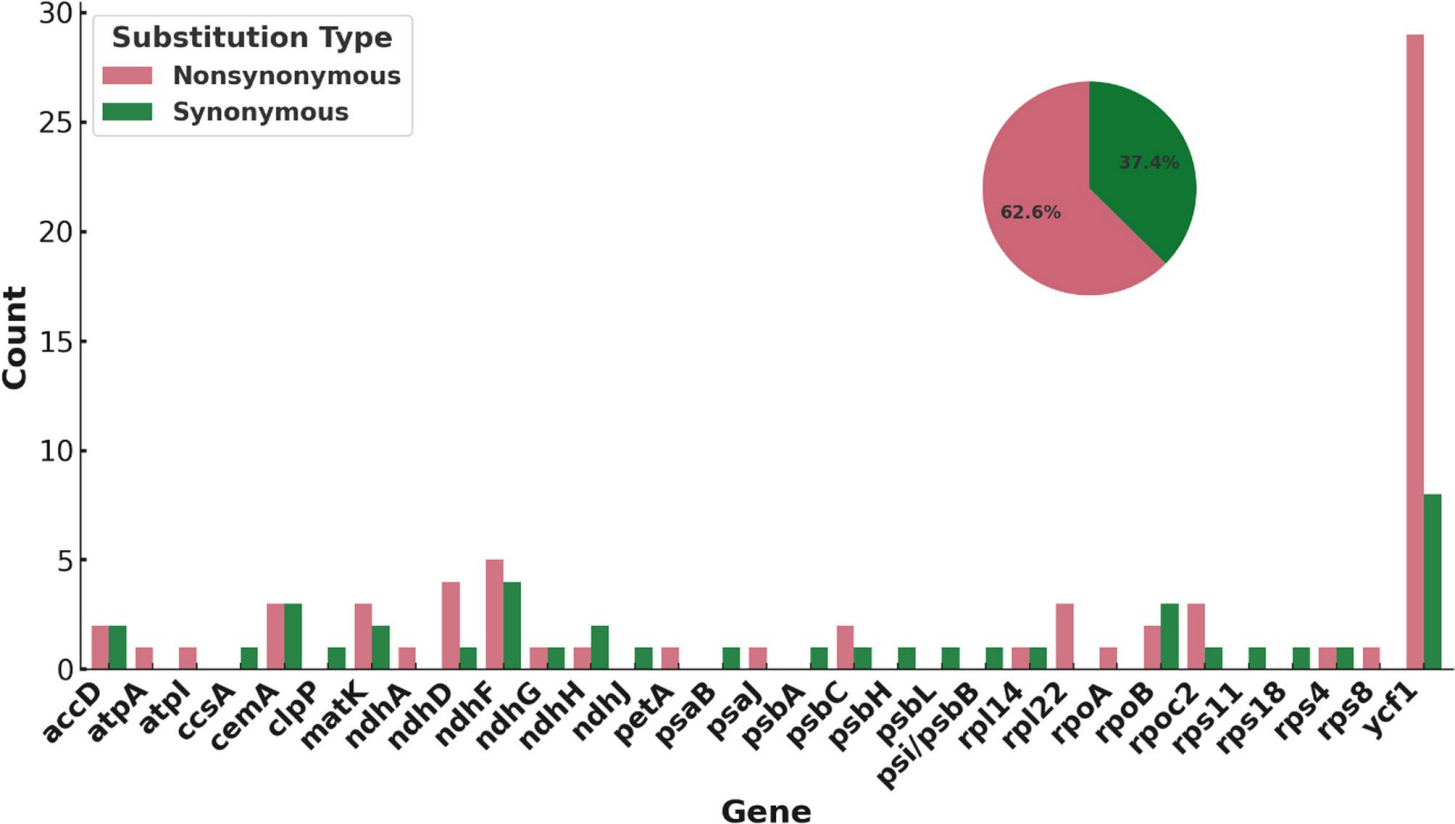
Distribution of synonymous and nonsynonymous substitutions in butternut and Japanese walnut. The bar chart shows the counts of synonymous (green) and nonsynonymous (red) substitutions across genes in butternut and Japanese walnut. The pie chart summarizes the overall proportions of substitution types

The analysis revealed notable codon and amino acid variations between butternut and Japanese walnut across 32 genes in their chloroplast genomes, comprising 37.4% synonymous and 62.6% nonsynonymous mutations (Fig. 4; Table 1; Supplementary Table 4). Five unique SNPs were identified in the *matK* gene. Two of the five SNPs have synonymous effects. Among the other three remaining substitutions, only one is a radical substitution, where glutamine is substituted for a lysine (CAA → AAA).

The *ycf1* gene showed the highest variability, with 37 unique SNPs resulting in 8 synonymous and 29 non-synonymous mutations. Fifteen of the 29 nonsynonymous mutations are considered radical and include GAT → TAT (aspartate to tyrosine), AAA → AAC (lysine to asparagine), and TTT → TTA (phenylalanine to leucine). According to our SNP analysis results for this gene, none of the amino acid substitutions were located in the alpha helix coding region of *ycf1*.

### Identification of CAPS marker candidates for matK and ycf1 genes

For CAPS markers, we focused on the *matK* and *ycf1* genes, which are consistently used for plant barcoding. For *matK*, 3 out of 5 SNPs were identified as suitable for CAPS design, while for *ycf1*, 9 out of 37 SNPs were selected. The predicted CAPS marker amplicons are typically shorter than 300 bp. When using the CAPS Finder tool, setting the nucleotide mismatch to zero provides greater flexibility in primer design. However, for SNP position 666 in *matK*, a mismatch count of two was required, resulting in the reverse primer overlapping the SNP. Primers designed for this SNP were predicted to amplify a 191 bp segment, and digestion with *BsaJI* in *J. ailantifolia* is expected to yield two fragments of 166 bp and 25 bp. For the remaining 11 predicted CAPS markers, the smallest expected fragment post-digestion is 79 bp (for SNP 117 in *matK*), ensuring that all fragments are within a detectable range for agarose gel analysis (Supplementary Table 6).

### Validation of CAPS and dCAPS markers by PCR and restriction enzyme

**Fig. 5:**
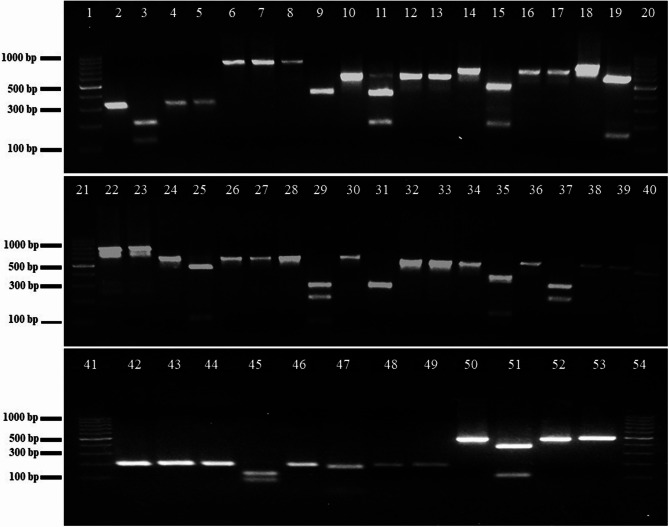
Restriction digestion profiles of candidate CAPS and dCAPS markers resolved on a 3% agarose gel. Lanes 1, 20, 21, 40, 41, and 54 contain the 100 bp DNA ladder (Thermo Scientific, cat. no. SM0241/2). For each marker, PCR products from *J. ailantifolia* and *J. cinerea* are shown before and after digestion with the corresponding restriction enzyme. For example, lanes 2 and 3 display CPS01-*ycf1* in *J. ailantifolia* before and after digestion, respectively, while lanes 4 and 5 show CPS01-*ycf1* in *J. cinerea*. This order was maintained for all subsequent markers. Lanes 2–39 represent CPS01-*ycf1* through CPS09-*ycf1* and lanes 42–53, CPS01-*matK* through CPS03-*matK*, as indicated. (Please see Supplementary Figure 7 for more information)

PCR amplification confirmed the expected amplicons for all primer pairs. Fragmentation analysis of 11 candidate CAPS markers and one dCAPS marker revealed that all markers generated the anticipated patterns in *J. ailantifolia* and *J. cinerea* (Fig. 5). The developed enzymes fully digested their respective amplicons in both *J. ailantifolia* and *J. cinerea*, except for the CPS03-*ycf1* amplicon in butternut, which PsiI-v2 partially digested, yielding three bands; nevertheless, the expected amplicon was observed, although the primary fragment remained intact. Still, the marker differentiated it from *J. ailantifolia*, which showed a single band. Additionally, the 25 bp DNA fragment produced by the dCAPS marker in *matK* (CPS02-*matK*) was clearly visible on the agarose gel (Fig. 5; Supplementary Table 7).

## Discussion

In this study, the chloroplast genome of butternut was assembled and annotated using paired-end data, which provided a comprehensive overview of its genetic architecture, revealing a highly conserved characteristic in terms of gene numbers and GC content that is consistent with other chloroplast genomes in the Juglandaceae family and other angiosperms​ [8, 30, 31]​.

### Comparative chloroplast genomics among *Juglan *species

Comparative analysis of the butternut chloroplast genome with those of Japanese walnut and black walnut reveals high similarity in gene content, indicating a conserved chloroplast genome structure across these species. However, notable structural variations and gene loss events provide insights into evolutionary divergence within the Juglandaceae family. A prominent structural feature involves the *ycf1* gene, which spans the IRb/SSC junction and extends into the SSC region. This configuration results in a truncated pseudogene copy (^Ψ^*ycf1*) at the IRb/SSC boundary, meaning a non-functional duplicated fragment exists alongside the complete functional gene. Our analysis of NCBI data shows that *ycf1* duplication is also present in other Asian *Juglans* species, including *J. cathayensis* (NC_033893.1), *J. mandshurica* (NC_033892.1), and *J. regia* (NC_028617.1), suggesting this is a conserved feature across the genus. The *ycf1* gene in both butternut and Japanese walnut is notably long (5,676 bp), encoding 1,891 amino acids. This aligns with findings that specific lineages encode *ycf1* proteins exceeding 1,500 amino acids, with some reaching over 3,000 amino acids [14]. Although the exact function of the *ycf1* protein remains unclear, it is crucial for plant viability [32] and has been implicated in photosynthesis. The gene has also been identified as a critical marker in phylogenetic studies [31, 33]. Our comparative analysis also identified the loss of *infA* and a pseudo copy of *ycf1* genes in butternut, Japanese walnut, and black walnut. Gene loss or pseudogenization in chloroplast genomes results in the inability to encode functional proteins [34]. However, the remaining gene sequences may retain regulatory functions similar to those found in other non-coding regions [35], and pseudogenes can play significant roles in both normal physiological and pathological conditions [36]. The loss of *infA* has been previously reported in the Liliaceae family, as documented by She et al. [37], with no apparent detrimental impacts, suggesting functional compensation through nuclear-encoded genes or alternative mechanisms. The *infA* gene encodes translation initiation factor 1 (IF1), a component involved in chloroplast protein synthesis, and is located near other soluble translation factor coding regions in the chloroplast genome [38]. Among bacterial genes encoding translation initiation factors, only *infA* has a homolog in the plastome of higher plants [39]. Similarly, *ycf1*, predicted as a pseudogene, has an unusual reading frame for plastid genes and is typically located at the junction of the IR and SSC regions [40]. These gene loss events and structural variations are crucial for understanding the evolutionary pressures and adaptations that have shaped the chloroplast genomes of these *Juglans* species.

### Butternut reveals distinct allele frequency patterns compared to Japanese walnut

Our allele frequency analysis revealed distinct patterns between butternut and Japanese walnut, with higher frequencies of alleles A, G, and C in butternut. This finding is consistent with the allele frequency patterns observed in other chloroplast genome studies, such as those conducted on pomegranate and Ficus species, where specific allele distributions have been linked to environmental adaptation and evolutionary history​ [31, 33]. These allele frequency differences highlight the importance of SNP analysis in understanding the genetic basis of phenotypic traits and environmental responses. Some genes, such as *matK* and *ycf1*, were highly variable in butternut and Japanese walnut. Of all the variations identified, 31.7% were located within CDS regions and found in most chloroplast genes (68%). Interestingly, 33% of that SNP variation was found in the CDS regions of only two genes, *ycf1*, and *matK*, with 84 and 40 SNPs, respectively. This high variability within these genes was previously reported in the chloroplast genomes of eastern Asian *Juglans* species [8].

### Codon and amino acid variations of butternut and Japanese walnut

Codon and amino acid variations in the chloroplast genomes of butternut and Japanese walnut provide critical insights into species differentiation and evolutionary dynamics. Genes such as *matK*, *atpA*, *rps4*, *rps8*, *rpl14*, *rpl22*, and *ycf1* exhibit both synonymous and non-synonymous mutations, which may potentially affect protein function and species-specific traits. The *ycf1* gene exhibits the highest variability, with 37 unique SNPs, making it a key marker for distinguishing between butternut and Japanese walnut, and is consistent to use as a barcode and for phylogenetic relationships in other plants [18]. Non-synonymous mutations in these genes suggest potential functional differences in the encoded proteins, which could contribute to distinct phenotypic traits. These variations are vital for species authentication, particularly in conservation efforts and hybridization studies, where precise identification is essential to clarify genetic lineages. Amino acid mutations are classified as conservative or radical, with radical substitutions more likely to affect protein stability [41]. Although amino acid substitutions can alter protein function, SNP analysis in this study revealed no substitutions within the alpha-helix region of *ycf1*, a region critical for maintaining the protein’s secondary and tertiary structure. This region comprises six alpha-helix domains (amino acids 106–326) that anchor the complex [14]. Additionally, key *Tic214* residues (K1939, K1880, T1795), which are critical for protein function, showed no variation. The *Tic214* protein plays a vital role in the TOC-TIC super-complex, facilitating chloroplast protein import by bridging the TOC and TIC complexes and spanning the intermembrane space [14].

### Application CAPS Markers for authentication of *Juglans* species

DNA barcoding uses short DNA sequences from organelle genomes to differentiate plant species and is widely applied for species-specific identification, biodiversity assessment, and evolutionary studies [42, 43]. Accurate identification is crucial, particularly when congeners with distinct interspecific traits (i.e., disease resistance and adaptive traits) can hybridize naturally. For example, hybridization between butternut and Japanese walnut over the past century has complicated the identification of genetic background, as morphological markers are often insufficient to distinguish between the species. This is especially important given the threat of BCD to butternut populations, highlighting the need for practical conservation tools. Chloroplast markers, with their high conservation, copy numbers, and sufficient interspecific divergence, are ideal for developing DNA barcodes for species verification [12, 43, 44]. In this study, we identified 32 species-specific SNPs in chloroplast coding sequences of butternut and Japanese walnuts. We identified and validated 12 candidate CAPS markers targeting *matK* and *ycf1* for these species using in silico and experimental approaches. These predicted markers provide a basis for future research on a wide range of germplasms and hold promise as tools for species authentication and conservation, especially for distinguishing among closely related Asian walnuts.

## Conclusion

This study comprehensively analyzed chloroplast genomes in butternut and Japanese walnut, revealing structural variations, SNP patterns, and differences in gene content. The assembled butternut chloroplast genome demonstrates high structure and GC content conservation, consistent with other Juglandaceae species. Comparative analyses identified 32 species-specific SNP markers, exhibiting significant variability in the *ycf1* and *matK* genes, which are important for species differentiation. The developed species-specific CAPS markers provide practical tools for species authentication and conservation, supporting efforts to protect butternut populations and mitigate the impacts of hybridization. These findings enhance understanding of chloroplast genome dynamics, supporting biodiversity assessment and conservation genetics.

## Supplementary Information

Supplementary Material 1.

Supplementary Material 2.

Supplementary Material 3.

Supplementary Material 4.

Supplementary Material 5.

Supplementary Material 6.

Supplementary Material 7.

## Data Availability

All the data included in the manuscript as a supplementary files and the genome deposited in NCBI: chloroplast genome of butternut ( J. cinerea, accession number: O13R4831).

## References

[1] Moerman DE. Native American ethnobotany. Portland: Timber; 1998.

[2] Renlund DW. Forest pest conditions in Wisconsin. In: Annual Report 1971. Madison: Wisconsin Department of Natural Resources; 1971. pp. 26–28.

[3] Environment Canada. Species at Risk Public Registry. 2010. Available from: https://www.canada.ca/en/environment-climate-change/services/species-risk-public-registry.html.

[4] Stritch L, Barstow M. *Juglans cinerea*, Butternut. The IUCN Red List of Threatened Species 2019: e.T62019689A62019696. 2019. Available from: https://www.iucnredlist.org/species/62019689/62019696.

[5] Hoban SM, Borkowski DS, Brosi SL, McCLEARY TS, Thompson LM, McLACHLAN JS, Pereira MA, Schlarbaum SE, ROMERO-SEVERSON, J.E.A.N.N.E. Range‐wide distribution of genetic diversity in the North American tree juglans cinerea: A product of range shifts, not ecological marginality or recent population decline. Mol Ecol. 2010;19(22):4876–91. 21040046 10.1111/j.1365-294X.2010.04834.x

[6] Skender A, Kurtovic M, Drkenda P, Becirspahic D, Ebrahimi A. Phenotypic variability of autochthonous walnut (*Juglans regia* L) genotypes Innorthwestern Bosnia and Herzegovina. Turkish J Agric Forestry. 2020;44(5):517–25.

[7] Ruhfel BR, Gitzendanner MA, Soltis PS, Soltis DE, Burleigh JG. From algae to angiosperms—inferring the phylogeny of green plants (Viridiplantae) from 360 plastid genomes. BMC Evol Biol. 2014;14:1–27. 10.1186/1471-2148-14-23 PMC3933183 24533922

[8] Zhou H, Hu Y, Ebrahimi A, Liu P, Woeste K, Zhao P, et al. Whole genome based insights into the phylogeny and evolution of the Juglandaceae. BMC Ecol Evol. 2021;21(1):1–16. 34674641 10.1186/s12862-021-01917-3 PMC8529855

[9] Sabir J, Schwarz E, Ellison N, Zhang J, Baeshen NA, Mutwakil M, et al. Evolutionary and biotechnology implications of plastid genome variation in the inverted-repeat-lacking clade of legumes. Plant Biotechnol J. 2014;12:743–54. 24618204 10.1111/pbi.12179

[10] Kane N, Sveinsson S, Dempewolf H, Yang JY, Zhang D, Engels JM, et al. Ultra-barcoding in Cacao (*Theobroma* spp.; Malvaceae) using whole Chloroplast genomes and nuclear ribosomal DNA. Am J Bot. 2012;99:320–9. 22301895 10.3732/ajb.1100570

[11] Mccleary TS, Robichaud RL, Nuanes S, Anagnostakis SL, Schlarbaum SE, ROMERO-SEVERSON, J.E.A.N.N E. Four cleaved amplified polymorphic sequence (CAPS) markers for the detection of the juglans ailantifolia Chloroplast in putatively native *J. cinerea* populations. Mol Ecol Resour. 2009;9(2):525–7. 21564682 10.1111/j.1755-0998.2008.02465.x

[12] Giang VNL, Waminal NE, Park HS, Kim NH, Jang W, Lee J, et al. Comprehensive comparative analysis of Chloroplast genomes from seven *Panax* species and development of an authentication system based on species-unique single nucleotide polymorphism markers. J Ginseng Res. 2020;44(1):135–44. 32148396 10.1016/j.jgr.2018.06.003 PMC7033337

[13] Eckardt NA. Genomic hopscotch: gene transfer from plastid to nucleus. Plant Cell. 2006;18(12):2865–8.

[14] de Vries J, Sousa FL, Bölter B, Soll J, Gould SB. *YCF1*: a green TIC. Plant Cell. 2015;27(7):1827–33. 25818624 10.1105/tpc.114.135541 PMC4531346

[15] Turmel M, Otis C, Lemieux C. The Chloroplast genome sequence of *Chara vulgaris* sheds new light into the closest green algal relatives of land plants. Mol Biol Evol. 2006;23(6):1324–38. 16611644 10.1093/molbev/msk018

[16] Stamatakis A. RAxML version 8: a tool for phylogenetic analysis and post-analysis of large phylogenies. Bioinformatics. 2014;30(9):1312–3. 24451623 10.1093/bioinformatics/btu033 PMC3998144

[17] Mohanta TK, Mishra AK, Khan A, Hashem A, Abd_Allah EF, Al-Harrasi A. Gene loss and evolution of the plastome. Genes. 2020;11(10):1133. 32992972 10.3390/genes11101133 PMC7650654

[18] Dong W, Xu C, Li C, Sun J, Zuo Y, Shi S, Cheng T, Guo J, Zhou S. ycf1, the most promising plastid DNA barcode of land plants. Sci Rep. 2015;5(1):1–5. 10.1038/srep08348 PMC4325322 25672218

[19] Ahmed I, Lockhart PJ, Agoo EM, Naing KW, Nguyen DV, Medhi DK, et al. Evolutionary origins of Taro (*Colocasia esculenta*) in Southeast Asia. Ecol Evol. 2020;10(23):13530–43. 33304557 10.1002/ece3.6958 PMC7713977

[20] Doyle JJ, Doyle JL. A rapid DNA isolation procedure for small quantities of fresh leaf tissue. Phytochem Bull. 1987;19:11–5.

[21] Ebrahimi A, Mathur S, Lawson SS, LaBonte NR, Lorch A, Coggeshall MV, et al. Microsatellite borders and micro-sequence conservation in *Juglans*. Sci Rep. 2019;9(1):3748. 30842460 10.1038/s41598-019-39793-z PMC6403238

[22] Jin JJ, Yu WB, Yang JB, Song Y, DePamphilis CW, Yi TS, et al. GetOrganelle: a fast and versatile toolkit for accurate de Novo assembly of organelle genomes. Genome Biol. 2020;21:1–31. 10.1186/s13059-020-02154-5 PMC7488116 32912315

[23] Lehwark P, Greiner S. GB2sequin: a file converter Preparing custom GenBank files for database submission. Genomics. 2019;111:759–61. 29842948 10.1016/j.ygeno.2018.05.003

[24] Tillich M, Lehwark P, Pellizzer T, Ulbricht-Jones ES, Fischer A, Bock R, et al. GeSeq—versatile and accurate annotation of organelle genomes. Nucleic Acids Res. 2017;45(W1):W6–11. 28486635 10.1093/nar/gkx391 PMC5570176

[25] Zhang BW, Xu LL, Li N, Yan PC, Jiang XH, Woeste KE, Lin K, Renner SS, Zhang DY, Bai WN. Phylogenomics reveals an ancient hybrid origin of the Persian walnut. Mol Biol Evol. 2019;36(11):2451–61. 31163451 10.1093/molbev/msz112

[26] Katoh K, Standley DM. MAFFT multiple sequence alignment software version 7: improvements in performance and usability. Mol Biol Evol. 2013;30(4):772–80. 23329690 10.1093/molbev/mst010 PMC3603318

[27] Li H. 2014. Toward better understanding of artifacts in variant calling from high-coverage samples. Bioinformatics. 2014;30:2843–2851. 10.1093/bioinformatics/btu356 PMC4271055 24974202

[28] Wysoker A, Tibbetts K, Fennell T. Picard tools version 1.90. 2013. Available from: https://github.com/broadinstitute/picard.

[29] McKenna A, Hanna M, Banks E, Sivachenko A, Cibulskis K, Kernytsky A, et al. The genome analysis toolkit: a mapreduce framework for analyzing next-generation DNA sequencing data. Genome Res. 2010;20(9):1297–303. 20644199 10.1101/gr.107524.110 PMC2928508

[30] Liu X, Luo J, Zhang M, Wang Q, Liu J, Wu D, Fu Z. Phylogenomic analysis of two species of parasenecio and comparative analysis within tribe senecioneae (Asteraceae). Diversity. 2023;15(4):563.

[31] Huang Y, Li J, Yang Z, An W, Xie C, Liu S, Zheng X. Comprehensive analysis of complete Chloroplast genome and phylogenetic aspects of ten ficus species. BMC Plant Biol. 2022;22(1):253. 35606691 10.1186/s12870-022-03643-4 PMC9125854

[32] Drescher A, Ruf S, Calsa T, Carrer H, Bock R. The two largest Chloroplast genome-encoded open reading frames of higher plants are essential genes. Plant J. 2000;22(2):97–104. 10792825 10.1046/j.1365-313x.2000.00722.x

[33] Singh NV, Patil PG, Sowjanya RP, Parashuram S, Natarajan P, Babu KD, et al. Chloroplast genome sequencing, comparative analysis, and discovery of unique cytoplasmic variants in pomegranate (*Punica granatum* L). Front Genet. 2021;12:704075. 34394192 10.3389/fgene.2021.704075 PMC8356083

[34] Prade VM, Gundlach H, Twardziok S, Chapman B, Tan C, Langridge P, et al. The pseudogenes of barley. Plant J. 2018;93(3):502–14. 29205595 10.1111/tpj.13794

[35] Poliseno L, Salmena L, Zhang J, Carver B, Haveman WJ, Pandolfi PP. A coding-independent function of gene and pseudogene mRNAs regulates tumor biology. Nature. 2010;465(7301):1033–8. 20577206 10.1038/nature09144 PMC3206313

[36] Lu X, Yang J, Zhao S, Zhang X, Meng Y, Li M, et al. Pseudogene RNA: a novel functional molecule in plants. Int J Mol Sci. 2015;16(11):25352–63.

[37] She R, Zhao P, Zhou H, Yue M, Yan F, Hu G, et al. Complete Chloroplast genomes of liliaceae (sl) species: comparative genomic and phylogenetic analyses. Nord J Bot. 2020;38(1):1–12.

[38] Sijben-Muller EA, Neuteboom LW, de Vries PM, van den Berg H. Translation initiation factor IF1 in Escherichia coli. Eur J Biochem. 1986;154(2):455–9.

[39] Millen RS, Olmstead RG, Adams KL, Palmer JD, Lao NT, Heggie L, Kavanagh TA, Hibberd JM, Gray JC, Morden CW, Calie PJ, Jermiin LS, Wolfe KH. Many parallel losses of infa from Chloroplast DNA during angiosperm evolution with multiple independent transfers to the nucleus. Plant Cell. 2001;13(3):645–58. 10.1105/tpc.13.3.645. 11251102 10.1105/tpc.13.3.645 PMC135507

[40] Raubeson LA, Jansen RK. Chloroplast genomes of plants. Plant diversity and evolution: genotypic and phenotypic variation in higher plants. Cambridge: Cambridge University Press; 2005. pp. 45–68.

[41] Zhang J. Rates of Conservative and radical nonsynonymous nucleotide substitutions in mammalian nuclear genes. J Mol Evol. 2000;50(1):56–68. 10654260 10.1007/s002399910007

[42] Zuo Y, Chen Z, Kondo K, Funamoto T, Wen J, Zhou S. DNA barcoding of *Panax* species. Planta Med. 2011;77(02):182–7. 20803416 10.1055/s-0030-1250166

[43] Luo X, Zhou H, Cao D, Yan F, Chen P, Wang J, Woeste K, Chen X, Fei Z, An H, Malvolti M. Domestication and selection footprints in Persian walnuts (*Juglans regia*). PLoS Genet. 2022;18(12):e1010513. 36477175 10.1371/journal.pgen.1010513 PMC9728896

[44] Nguyen VB, Park HS, Lee SC, Lee J, Park JY, Yang TJ. Authentication markers for five major *Panax* species developed via comparative analysis of complete Chloroplast genome sequences. J Agric Food Chem. 2017;65(30):6298–306. 28530408 10.1021/acs.jafc.7b00925

