# Supplementary material for: Comparative analysis of butternut (Juglans cinerea) and Japanese walnut (Juglans ailantifolia) chloroplast genomes
Source: BMC Plant Biol. 2025 Dec 8;26:68. doi: 10.1186/s12870-025-07678-1 (PMC12797841; doi:10.1186/s12870-025-07678-1)
Supplement: Supplementary file 6 — Supplementary Material 6. [file 12870_2025_7678_MOESM6_ESM.docx]

**Supplementary Table 6.** Sequences of potential CAPS marker primers designed for the matK and ycf1 genes. The table includes the start and end positions of the amplicons, suitable restriction enzymes, and SNP positions. In the "Fragments After Digestion" columns, each slash (/) represents a restriction site of the corresponding enzyme within the amplicon. The numbers before and after the slash indicate the expected fragment sizes resulting from amplicon digestion. JC refers to *J. cinerea*, JA refers to *J. ailantifolia*. ^&^ **dCAPS (derived Cleaved Amplified Polymorphic Sequence)** markers are primers designed with intentional mismatches near SNP sites to create a restriction enzyme recognition site that does not naturally occur. This allows discrimination between alleles even when the SNP does not make a native restriction site.

| Markers | Primer’s sequence | Gene | Start  Position  amplicon | End  Position  amplicon | Amplicon length | SNP position in CDS | Restriction enzyme | Fragments in JC after digestion | Fragments in JA after digestion |
| --- | --- | --- | --- | --- | --- | --- | --- | --- | --- |
| CPS01-matk | F: GGCAACATGACTTCCTATACCCACT | matK | 41 | 236 | 196 | 117 | *Bgl*II | 79/117 | 196 |
|  | R:TGGTTAGAATCATTAGTGGAAATCAGCA |  |  |  |  |  |  |  |  |
| CPS02-matk ^&^ | F: TGAAAGATGCCTCCTCTTTGCAT | matK | 500 | 691 | 191 | 666 | BsaJI | 191 | 166/25 |
|  | R: GAGAAGATTGTTTACGGAGACCAAG |  |  |  |  |  |  |  |  |
| CPS03-matk | F: CTAAATACTTCAATGGTGCGGAGTC | matK | 1015 | 1503 | 489 | 1398 | BsaJI | 489 | 109/379 |
|  | R: CCGCCCATTGATGCAAATAATATCT |  |  |  |  |  |  |  |  |
| CPS01-ycf1 | F: TCATACGTGTMAAAGTGATGGA | YCF1 | 1170 | 1500 | 330 | 1375 | *Xba*I | 330 | 204/126 |
|  | R: CGAAAGGGTCCGTTAAATAAAGAAT |  |  |  |  |  |  |  |  |
| CPS02-ycf1 | F: TCATACGTGTMAAAGTGATGGA | YCF1 | 1170 | 1953 | 783 | 1542 | *Esp3*I | 377/406 | 783 |
|  | R: TCTTCAGTTGCTACTTCCTGTT |  |  |  |  |  |  |  |  |
| CPS03-ycf1 | F: CCTTTACGTATCCRCCAAGTTTATC | YCF1 | 1205 | 1758 | 553 | 1578 | *Psi*I | 553 | 374/179 |
|  | R: TCCGGAAATAAATAAAGTCCTTTCAA |  |  |  |  |  |  |  |  |
| CPS04-ycf1 | F: GTCCGGAGAATACCGATACTAATG | YCF1 | 2018 | 2664 | 646 | 2473 | *Dde*I | 646 | 456/190 |
|  | R: TGAATACCGTCTSTTAACCAGTTT |  |  |  |  |  |  |  |  |
| CPS05-ycf1 | F: GAGGTGGCTTTGATACGTTATTC | YCF1 | 2074 | 2813 | JC:739 | 2651 | *Hpa*I | 739 | 575/155 |
|  | R: AAGGTAATTCAGTTTCCATTCCC |  |  |  | JA:730 |  |  |  |  |
| CPS06-ycf1 | F: CCATAATCAAATTATTCCYGAATCGTC | YCF1 | 3114 | 3693 | 579 | 3225 | *Psi*I | 579 | 113/466 |
|  | R: CTGACTTGGATTTGTGAGAATTTGTAG |  |  |  |  |  |  |  |  |
| CPS07-ycf1 | F: CCATAATCAAATTATTCCYGAATCGTC | YCF1 | 3114 | 3693 | 579 | 3489 | DraI | 288/291 | 288/87/204 |
|  | R: CTGACTTGGATTTGTGAGAATTTGTAG |  |  |  |  |  |  |  |  |
| CPS08-ycf1 | F: CTCGAGAGATAAATATTAGCCAGACC | YCF1 | 3275 | 3800 | 525 | 3659 | *Nde*I | 383/142 | 525 |
|  | R: CGGAATCAAATATTCCTTGCGTT |  |  |  |  |  |  |  |  |
| CPS09-ycf1 | F: TCTCACAAATCCAAGTCAGTAAC | YCF1 | 3674 | 4218 | 544 | 3900 | *Xba*I | 544 | 225/319 |
|  | R: AGTTTACGTTTAGSCGTATTGT |  |  |  |  |  |  |  |  |
