# Supplementary material for: Comparative analysis of butternut (Juglans cinerea) and Japanese walnut (Juglans ailantifolia) chloroplast genomes
Source: BMC Plant Biol. 2025 Dec 8;26:68. doi: 10.1186/s12870-025-07678-1 (PMC12797841; doi:10.1186/s12870-025-07678-1)

**Original unprocessed Agarose Gel images related to Figure 5**

**Title:**
Full-length, unprocessed agarose gel images corresponding to Figure 5 of the manuscript:
“Comparative Analysis of Butternut (*Juglans cinerea*) and Japanese Walnut (*Juglans ailantifolia*) Chloroplast Genomes.”

**Figure 5 – Validation of CAPS Marker by PCR and Restriction Enzyme Digestion**

The following panels present the original, uncropped agarose gel images used to generate the composite Figure 5 in the main manuscript. Each gel represents independent PCR amplifications and CAPS enzyme digestions used for marker validation. Molecular size markers (ladders) are visible on both sides of each gel. All images were captured directly from the gel documentation system, with no post-processing other than minor contrast adjustment for visualization in the published figure.

| Panel | Original Image File Name | Description | Lanes Included | Relation to Published Figure |
| --- | --- | --- | --- | --- |
| (a) | 1-20.jpg | PCR + restriction digest results for samples 1–20 | Lanes 1–20 | Top section of composite Figure 5 |
| (b) | 21-40.jpg | PCR + restriction digest results for samples 21–40 | Lanes 21–40 | Middle section of composite Figure 5 |
| (c) | 41-54.jpg | PCR + restriction digest results for samples 41–54 | Lanes 41–54 | Bottom section of composite Figure 5 |

**Original gel images:**

**(a)** Unprocessed agarose gel image
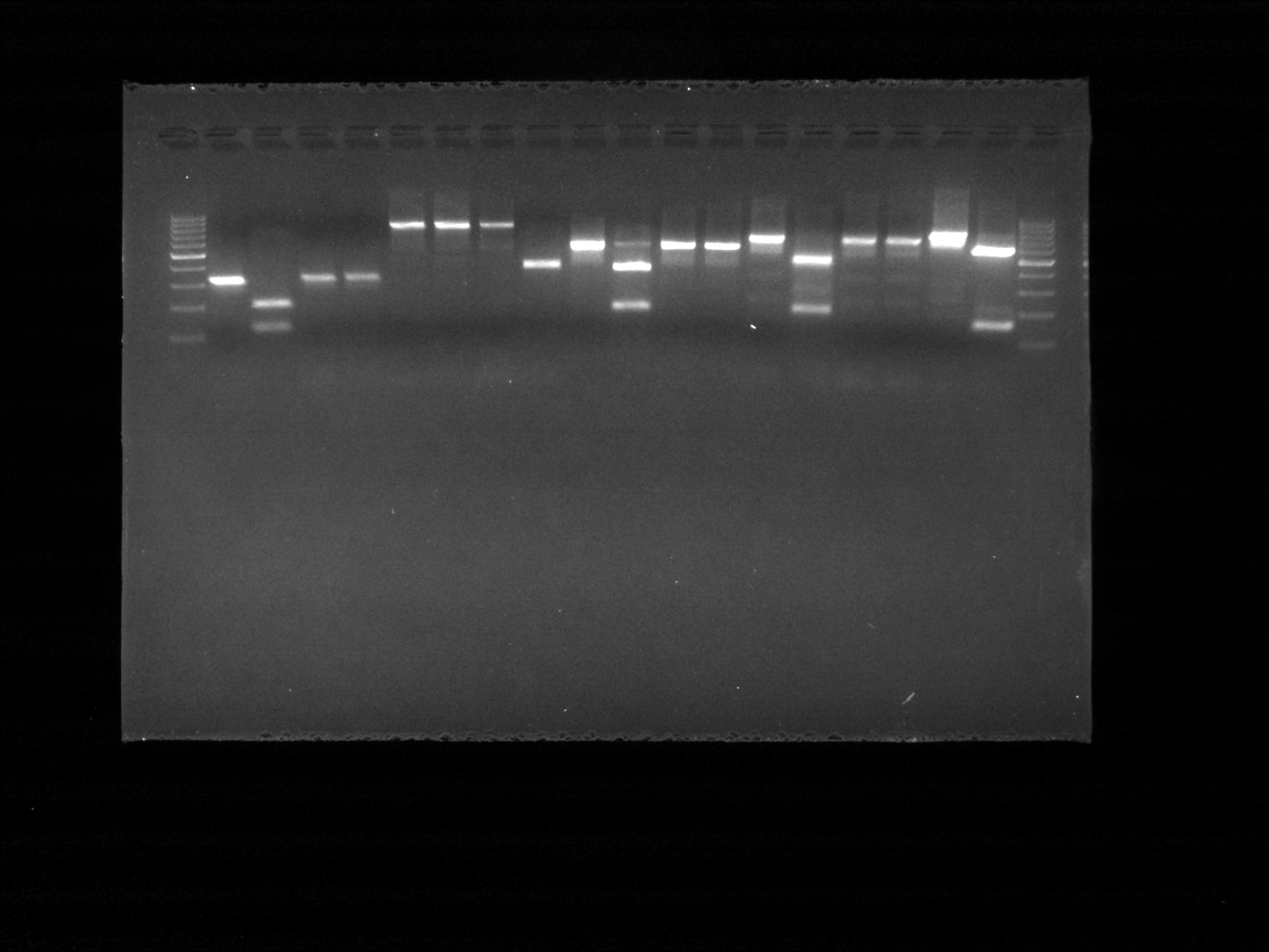


**(b)** Unprocessed agarose gel image
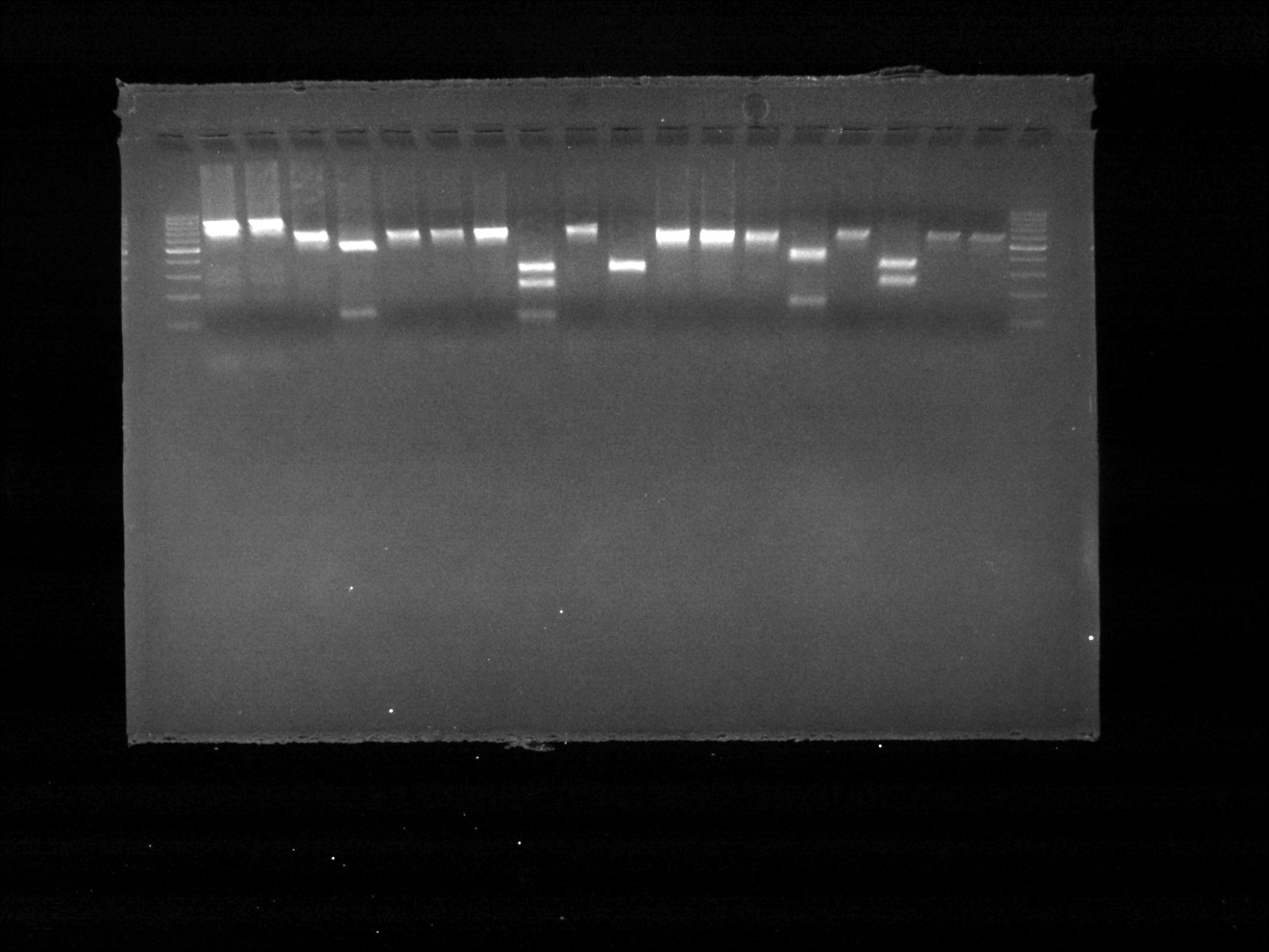


**(c)** Unprocessed agarose gel image
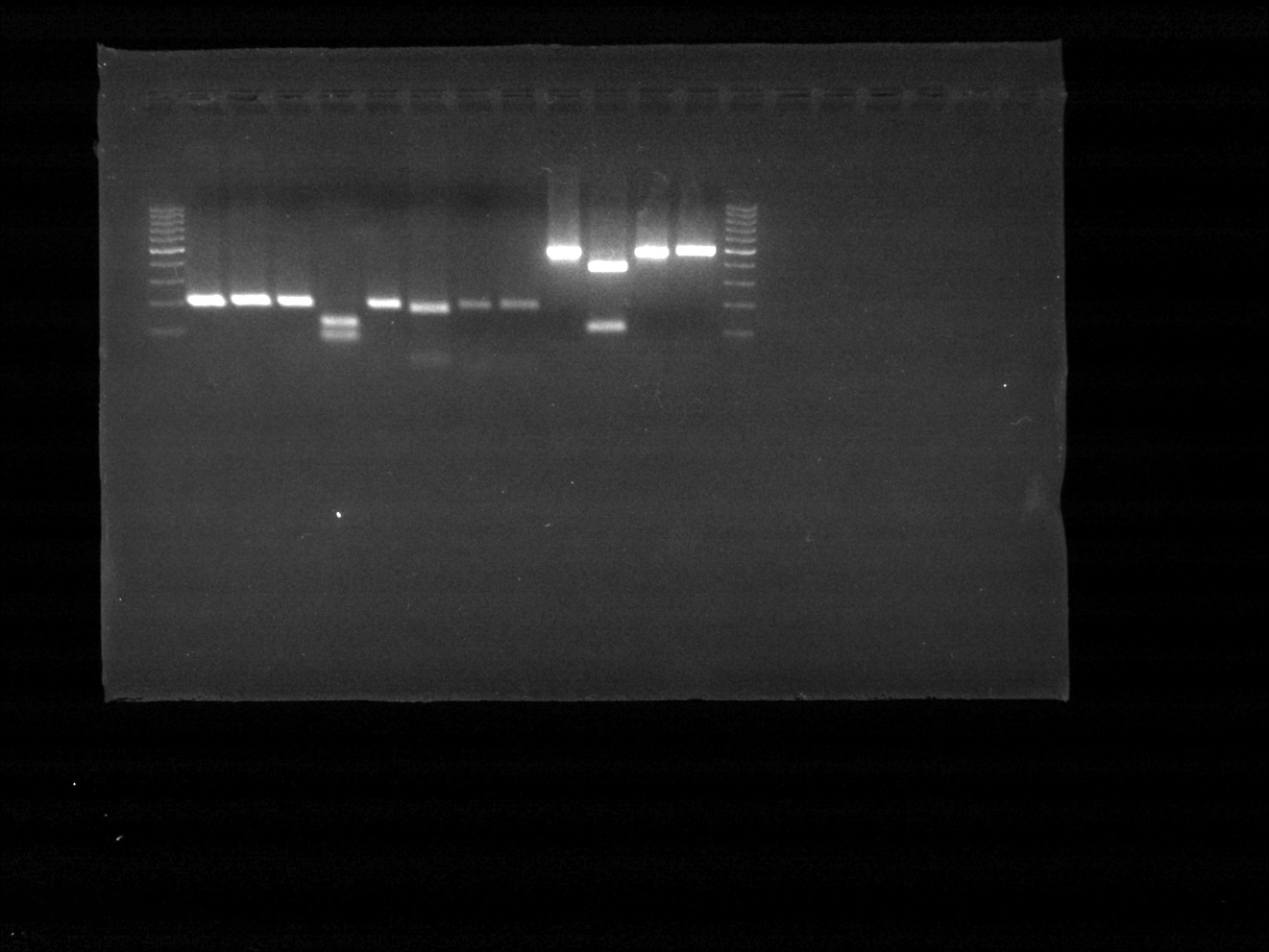

Supplement: Supplementary file 7 — Supplementary Material 7. [file 12870_2025_7678_MOESM7_ESM.docx]
